# Participation of Low Molecular Weight Electron Carriers in Oxidative Protein Folding

**DOI:** 10.3390/ijms10031346

**Published:** 2009-03-20

**Authors:** Éva Margittai, Miklós Csala, József Mandl, Gábor Bánhegyi

**Affiliations:** Department of Medical Chemistry, Molecular Biology and Pathobiochemistry, Semmelweis University & MTA-SE Pathobiochemistry Research Group, 1444 Budapest, POB 260, Hungary; E-Mails: eva.margittai@eok.sote.hu (E.M.); miklos.csala@eok.sote.hu (M.C.); jozsef.mandl@eok.sote.hu (J.M.)

**Keywords:** Oxidative folding, ascorbate, tocopherol, vitamin K, protein disulfide isomerase, Ero1, endoplasmic reticulum, glutathione, small-molecule catalysts

## Abstract

Oxidative protein folding is mediated by a proteinaceous electron relay system, in which the concerted action of protein disulfide isomerase and Ero1 delivers the electrons from thiol groups to the final acceptor. Oxygen appears to be the final oxidant in aerobic living organisms, although the existence of alternative electron acceptors, e.g. fumarate or nitrate, cannot be excluded. Whilst the protein components of the system are well-known, less attention has been turned to the role of low molecular weight electron carriers in the process. The function of ascorbate, tocopherol and vitamin K has been raised recently. *In vitro* and *in vivo* evidence suggests that these redox-active compounds can contribute to the functioning of oxidative folding. This review focuses on the participation of small molecular weight redox compounds in oxidative protein folding.

## 1. Introduction

**Figure 1.**
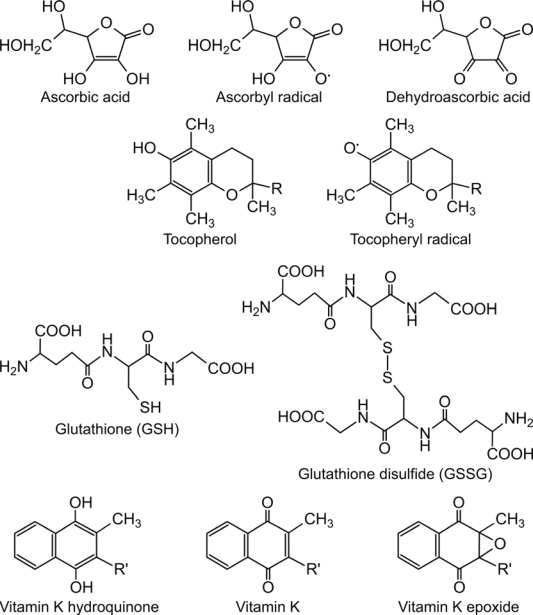
The chemical structure of small-molecule catalysts of oxidative protein folding. The structure of physiologically relevant electron carriers is shown. For the artificial small-molecules in the catalysis of oxidative protein folding see [48]. R = three isoprene units, R’ = variable number of unsaturated isoprenoid residues.

In mammalian cells, proteins that are directed to the plasma membrane, to endomembranes, or which will be secreted from the cell, are synthesized on the ribosomes of the rough endoplasmic reticulum (ER). After synthesis, these proteins are transferred to the lumen of the ER, where their native conformation evolves by several co-/post-translational modifications, e.g. the disulfide bond formation [1]. The oxidation of thiol groups to disulfide bond on a nascent protein is heavily dependent on the oxidative environment of the ER lumen, which has a much higher thiol redox potential with respect to the cytosol [2]. The generation and the maintenance of this oxidative environment is ensured by a microsomal electron transfer chain, which transfers the electrons from the thiol groups of newly synthesized proteins to a final electron acceptor, which would be the oxygen. The protein components of this transfer chain have been subject to extensive research. A functional connection between the two main protein components, the FAD-containing enzyme ER oxidoreductase 1 (Ero1), and the protein disulfide isomerase (PDI) has been well known for a long while; oxygen, the final electron acceptor can oxidize Ero1, which oxidizes PDI. Then PDI contributes to the formation of disulfide bonds of newly synthesized proteins by oxidizing the thiol groups [1]. The less known part of the process is the oxidation of Ero1, where beside oxygen and FAD, other small molecules contribute to the delivery of electrons, and thus in the maintenance of ER oxidative environment. However, these redox active small molecules have always been out of focus. This review will concentrate on the currently known small molecular weight, non-protein electron carriers of the microsomal electron transfer chain (Figure 1).

Recent studies have indicated the role of vitamin C (ascorbate) and vitamin E (tocopherol), the most abundant water-soluble and lipid-soluble antioxidants in the ER, in oxidative protein folding. Both antioxidants have two transmittable electrons, but are also able to donate only one electron. In this case free radicals - ascorbyl radical and vitamin E radical - are formed. Vitamin E radical is generated e.g. when a reactive oxygen species (ROS) oxidizes tocopherol. The redox connection between ascorbate and vitamin E is well-known; the tocopheryl radical can be re-reduced to vitamin E by ascorbate, while ascorbyl radical is produced [3]. Ascorbyl radical might be also generated upon the reaction of ascorbate directly with ROS. The ascorbyl radical – by the delivery of another electron – can disproportionate to dehydroascorbic acid, the completely oxidized form of ascorbate. Dehydroascorbic acid, unless re-reduced to ascorbate by glutathione, is susceptible for degradation. The degradation products recycle into the carbohydrate metabolism [4–5].

Ascorbate is synthesized in the hepatocytes of most animals [6]. The synthesis starts from UDP-glucose, and is regulated at the last step, which is catalyzed by the ER membrane-bound gulonolactone oxidase (GLO). The activity of this enzyme is controlled at the level of gene expression [7–8]. Interestingly, in humans and in few animal species (e.g. in guinea pig) GLO is inactive due to a mutation in the active site; hence these species need to take vitamin C with the diet [9].

## 2. Ascorbate-dependent protein thiol oxidation in the ER

In intact cells, the oxidative environment in the ER enables disulfide bond formation in newly synthesized proteins. It is, however, no longer the case in ER-derived microsomal vesicles, which suggests that the process needs a cytosolic factor or a membrane-permeable compound, which is lost during the preparation of microsomes. GSSG was long considered to have a key role in the process, but this assumption was conquered, since it was shown that the disulfide bond formation in GSH deficient yeast is intact [10], and the GSSG transport through the ER membrane is negligible [11]. Next candidate to fulfill this role was ascorbate, the most abundant water-soluble antioxidant in the ER. To execute its role as a pro-oxidant, ascorbate has to be present in its fully oxidized form, dehydroascorbic acid. While in plants the enzymes producing ascorbyl free radical are well-known [12], in animal tissues the oxidation of ascorbate was considered to be a non-enzymatic process catalyzed by metal ions, free radicals etc. However, lately ascorbate oxidase enzyme activity was detected at the surface of the ER [13].

In the presence of rat liver microsomes, ascorbate was continuously transformed to ascorbyl free radical and then dehydroascorbic acid. When microsomes were incubated in a cytosol like concentration of ascorbate at 37°C, concentration of ascorbate was decreasing with a constant rate during the incubation. Dehydroascorbic acid was detectable after a few minutes and its level remained constant as long as ascorbate was present in the medium. As it was detected by electron spin resonance spectroscopy, when ascorbate was incubated in an adequate buffer without microsomes, the typical ascorbyl free radical signal was present. In the presence of microsomes, a much higher ascorbyl radical signal was detected, which remained on the same level during the whole time of the experiment. As both ascorbyl free radical and dehydroascorbic acid are very instable, the maintenance of their constant level could be ensured only by a continuous ascorbate oxidation [13].

The fact that the addition of microsomes to the incubation medium radically increased ascorbate oxidation implied that there is a microsomal ascorbate oxidase activity. Pretreatment of microsomes with heat or protease significantly decreased the ascorbyl radical production and almost completely eliminated the ascorbate oxidase activity, indicating that ascorbate oxidation was indeed a protein mediated process.

Further information was gathered about the ascorbate oxidase enzyme by the addition of different inhibitors. Among several types of inhibitors, only various metal chelators reduced significantly the ascorbyl free radical production in the presence of microsomes. Among the chelators, the copper-specific neocuproine [14] caused the strongest effect, which is notable in the light that the ascorbate oxidase in plants contains copper [15].

In line with the consumption of the added ascorbate to liver microsomes, the oxidation of protein thiols is also observable, while without ascorbate addition remains negligible. The two processes – ascorbate consumption and thiol oxidation - showed good correlation with each other. In the presence of neocuproine both the ascorbate oxidation and the thiol oxidation were decreased [13].

**Figure 2.**
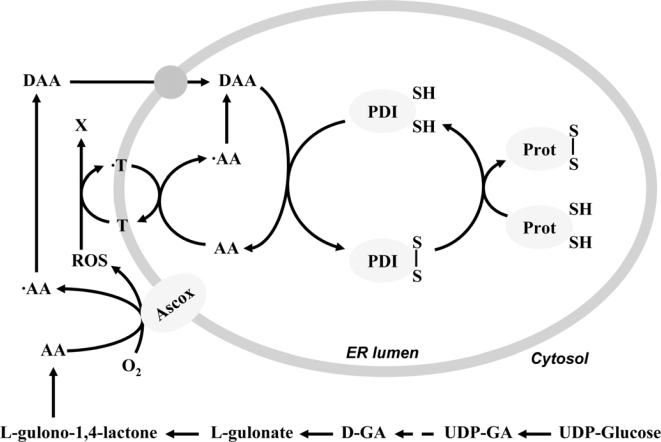
The role of ascorbate and tocopherol in the oxidative protein folding process. Ascorbate (AA) synthesized from UDP-glucose through UDP-glucuronic acid (UDP-GA) and glucuronate (GA) is oxidized to ascorbyl radical (·AA) by an unidentified ascorbate oxidase enzyme (Ascox) on the outer surface of the ER membrane. Further oxidation or dismutation yields dehydroascorbic acid (DAA), which can be transported into the ER lumen and oxidize the active thiols of protein disulfide isomerase (PDI) and hence contribute to the generation of disulfide bonds in the nascent proteins (Prot). The ROS produced by Ascox drives the luminal oxidation of AA and consequently further disulfide formation. The role of tocopherol (T) as a putative transmembrane electron carrier can be supposed in this phenomenon.

Since the thiol oxidation was coupled with the ascorbate oxidation, the role of dehydroascorbic acid in the process had to be supposed. Upon addition of dehydroascorbic acid, protein thiols were indeed oxidized, but dehydroascorbic acid proved to be less effective than ascorbate itself in this respect. This had a particular importance knowing the fact that, among the ascorbate/dehydroascorbic acid redox couple, the oxidized dehydroascorbic acid is the transported form through the ER membrane (Figure 2), while the transport of the reduced ascorbate is insignificant [16]. Accordingly, inhibition of the ascorbate oxidase enzyme detained the ascorbate transport to the lumen. Dehydroascorbic acid, as a small molecular weight electron acceptor can participate in the machinery of oxidative folding of proteins in the ER lumen. Dehydroascorbic acid was shown to be a substrate for the intraluminal PDI [17]. Dehydroascorbic acid can be reduced by PDI, oxidizing the dithiols on the enzyme. Oxidized PDI reacts with nascent proteins containing thiol groups, yielding protein disulfides, while the enzyme itself regenerating to its catalytically active form. As a result of this enzymatic function, ascorbate is generated (and accumulated) in the ER lumen (Figure 2). In certain pathological states, such as diabetes mellitus, unfolded nascent proteins accumulate in the ER lumen. The increased protein thiol availability under such circumstances results in an enhanced accumulation of luminal ascorbate, providing a further evidence for the process [18].

Based on the *in vitro* observations, that ascorbate might have a role in oxidative protein folding, experiments were designed to show its role also under *in vivo* conditions. In cells, the defective oxidative protein folding results in the accumulation of immature proteins, which in turn causes ER stress and unfolded protein response (UPR).

Since one of the most important functions of the ER is the synthesis and posttranslational modification of secretory and membrane proteins, the lumen of the organelle is equipped with a powerful protein-folding machine composed of chaperones, foldases and also with sensors that detect the presence of misfolded or unfolded proteins. Physiological and pathological effects or experimental agents that disturb the normal folding process provoke the UPR, an intracellular signaling pathway that coordinates ER protein-folding demand with protein-folding capacity and is essential to adapt to homeostatic alterations (collectively named as ER stress) that cause protein misfolding. These include changes in intraluminal calcium, altered glycosylation, nutrient deprivation, pathogen infection, expression of folding-defective proteins, and changes in the redox status. The principal events of ER stress and the UPR has been summarized in numerous recent reviews (see e.g. [19]).

The complex defense mechanism of UPR includes the up-regulation of ER chaperones (e.g. GRP78, GRP94) and foldases (e.g. PDI, ERP72) and the inhibition of protein synthesis. When these mechanisms are not able to rescue the cell from the stress situation, apoptosis is initiated to eliminate the diseased cell. Guinea pigs, which are unable to synthesize ascorbate, were used to model the effect of ascorbate deficiency on protein maturation *in vivo* [20]. Experiments were performed on the liver of the guinea pigs, which were kept on an ascorbate-free diet for four weeks. From the second week of the diet, there was no detectable amount of ascorbate in the liver, hence the diet was effective. The level of the lipid peroxidation, which is the mark of oxidative damage, elevated from the fourth week of the ascorbate-free diet, so the absence of the antioxidant function of ascorbate occurred at the end of diet. Among the markers of ER stress, in case of GRP78, GRP94 and PDI a gradual induction was found from the third week. Examining the apoptosis on histological liver slides, the apoptotic index was found to be increased in the livers derived from guinea pigs kept on ascorbate-free diet for three or four weeks. However, these changes were reversible, if after two weeks of ascorbate-free diet the animals were set back to a normal diet. In this case, the apoptosis and the lipid peroxidation were normalized and the levels of chaperones decreased to the control rate. These findings suggested that ascorbate participates not only in the collagen synthesis, as it was known long ago, but it also has a role in general protein maturation [20].

## 3. Role of lipophilic vitamins - vitamin E and K - in ascorbate-dependent protein thiol oxidation

The observation that ascorbate addition to microsomes promoted the protein thiol oxidation more effectively than dehydroascorbic acid foreshadowed that the formation of dehydroascorbic acid from ascorbate contributes to disulfide bond generation also in an indirect way. This suggested the involvement of an ER-membrane located lipid-soluble molecule, which connects the two processes: ascorbate oxidation on the outer surface, and protein thiol oxidation in the lumen.

Among the lipophilic electron carriers, the role of vitamin K was brought on earlier. The reduced form of vitamin K has a crucial role in the γ-carboxylation of proteins which require γ-carboxylated glutamate to evolve their activity. During γ-carboxylation, vitamin K is epoxidated, which then has to be reduced in order to regain its activity. The enzyme responsible for the reactivation is an integral ER membrane protein facing to the lumen, called vitamin K epoxide reductase (VKORC1) [21]. VKORC1 harbors a thioredoxin-like CXXC motif, which later turned out to be the redox active functional center of the enzyme. PDI was suggested to donate electrons to VKORC1 together with the thioredoxin system long ago. It has been shown that the addition of PDI to the thioredoxin system (thioredoxin + thioredoxin reductase + hydrogen donor) can improve the reduction of the vitamin K epoxide, replacing exquisitely the dithiothreitol cofactor and vitamin K reductase in bovine liver microsomes [22]. These data suggested that under *in vitro* conditions the formation of disulfide bonds was linked to the vitamin K-dependent γ-carboxylation of glutamate residues and the PDI/thioredoxin system might serve as the donor of the reducing equivalents in the vitamin K cycle. Later further evidences were lined up to show the link between the PDI dependent oxidative folding and the vitamin K reduction. Reduced protein substrates increased the rate of vitamin K reduction and γ-carboxylation of an artificial substrate, as it was shown in a recent study. Furthermore, a stable complex of PDI and VKORC1 has been also suggested [23]. These data demonstrated that PDI provides the electrons for reducing the CXXC group of VKORC1, so finally the oxidative protein folding might cover the energy needed for γ-carboxylation. The possibility that the reactions are also coupled *in vivo* has to be examined further.

Besides vitamin K, the role of tocopherol, the most abundant lipid-soluble antioxidant in the ER was also emerged in oxidative protein folding, and was proved by experiments performed on liver microsomes of tocopherol deficient rats [24]. The vitamin E content of the liver derived from these animals is hardly detectable; however, after *in vitro* addition of vitamin E the microsomal tocopherol level could be almost normalized. It was shown that in vitamin E deficient microsomes the intra- and extraluminal redox processes were partly uncoupled: while the intraluminal thiol oxidation decreased, the extraluminal ascorbate oxidation itself increased. Both effects were avoidable by *in vitro* tocopherol addition. The higher ascorbate oxidation was accompanied by an increased lipid peroxidation, which was probably caused by ROS produced on the extraluminal surface during ascorbate oxidation (Figure 2). Different water-soluble antioxidants were proved to be ineffective in eliminating ROS, which suggests that it was produced in the intimate closeness of the membrane, hence it is accessible only for the lipid-soluble antioxidants, such as vitamin E. So it was supposed that vitamin E might mediate the oxidative effect of ROS into the luminal thiols, and finally the oxidative power of ROS can get on the luminal protein thiols instead of the membrane lipids [24].

## 4. Final electron acceptor(s) of the protein folding system

In bacteria, oxidative protein folding occurs via Dsb proteins [25–26]. The disulfide bond formation takes place in the periplasmic region, which corresponds to the oxidative environment of the ER in mammals. DsbA, the direct catalyst of folding is present in this area. After disulfide bonds are formed, electrons flow from DsbA to an inner membrane protein, DsbB. DsbB passes electrons through ubiquinones to cytochrome oxidases, which use oxygen as the final electron acceptor. However, in the absence of oxygen, oxidative protein folding works exquisitely in bacteria. Under anaerobic conditions, alternative final acceptors, such as fumarate, nitrate or DMSO, can receive the electrons through menaquinone.

In yeast and mammals, the last protein component of the system, Ero1p also uses oxygen as electron acceptor [27]; *in vitro* assays demonstrated that during catalysis, Ero1p generates H_2_O_2_ in equimolar amounts to the number of disulfides formed [28]. It is questionable whether the eukaryotic cells are able to generate disulfides under anaerobic conditions, and in that case, which molecule can take the part of oxygen as the final electron acceptor. The *ero1-1* mutation in yeast causes temperature sensitivity under either aerobic or anaerobic conditions [29–30], which suggest that Ero1p is required for disulfide bond formation under both conditions; thus it seems feasible that beside oxygen, other electron acceptors are also able to reoxidize Ero1p. Indeed, elegant *in vitro* experiments performed under anaerobic conditions demonstrated, that Ero1p is able to transfer reducing equivalents to free flavins (FAD, FMN) and also to nonflavin molecules – among which two hem binding proteins (cytochrome *c*, cytochrome *b5*) and one protein with a copper-center (azurin) were examined successfully [28]. Free flavins are known to be present in the ER while the metalloproteins examined in the paper are not. Nevertheless, the experiments raise the possibility that Ero1p have other electron acceptors which might have physiological relevance under anaerobic conditions.

## 5. Artificial small-molecules in the catalysis of oxidative protein folding

Although recent studies have revealed that an ensemble of enzymes, chaperones, and small molecules are involved in the oxidative protein folding, still PDI remained at the center of the process. PDI, a 57 kDa molecule that resides in the ER of eukaryotic cells, is a member of the thioredoxin family of proteins [31–32]. This family is characterized by the ability to catalyze thiol-disulfide interchange reactions. Proteins of the family share a common Cys-Xaa-Xaa-Cys motif (CXXC, where X refers to any amino acid) in their active site, from which PDI has two CGHC sequences in its catalytic domains. The efficiency of these thiol-disulfide oxidoreductases is governed by two factors.

1. One is the reduction potential (E°′) of the disulfide bond which is formed between the Cys residues in the active site [33]. This value refers to the stability of the disulfide bond; the more stabile the disulfide, the lower the E°′. For PDI the E°′ is −180 mV, which corresponds to the redox potential of the ER lumen and allows PDI to remain in a nearly 50–50% mixture of its reduced and oxidized form (Table 1.).
2. The other factor that governs the efficiency of thiol-disulfide oxidoreductases is the acid dissociation constant (K_a_) of the N-terminal thiol group in the active site. Sulfhydryl group of free cysteine has a relatively high pK_a_ (8,5) and as a consequence it is relatively inert for redox reactions in physiological conditions. In contrast, some structural folds in thiol-disulfide oxidoreductases provide appropriated environments for changing the pK_a_ values of sulfhydryl groups. If this constant is close to the pH of the solution, a large part of the Cys can be deprotonated and stabilized in the anionic form called thiolate (RS^−^). The formed thiolate can initiate nucleophile attack to compose disulfide bonds in the substrate proteins. The pK_a_ value of Cys in the active site depends on the proteinaceous environment; e.g. in PDI the pK_a_ value of this Cys is 6.7 [34], which is low enough to result in a high amount of PDI-thiolate.

Additionally, having two thiol groups instead of one is also advantageous. If the mixed disulfide between the catalyst and the substrate persists too long, the second thiol can provide an escape route by removing the enzyme, thus preventing a long-lived, nonproductive bond [35].

As seen on the example of PDI, the properties of having two thiol groups, low E°′ and low thiol pK_a_ are important advantages of a protein folding catalyst. By using these criteria, several attempts aim at the production of an effective small molecule mimics PDI. The demand for producing such a small molecule rose from the fact that growth of disulfide-containing proteins in bacteria often results the formation of protein aggregates – called inclusion bodies – instead of the native folded protein [36]. These aggregates need to be resolubilized and folded *in vitro*, which is the most challenging step of the synthesis, since the commonly used glutathione redox buffer is hardly effective, and the usage of enzymes such PDI arises several problems.

A group of small molecule folding catalysts were designed by modeling the active-site of PDI. The linear CXXC peptides mimic the active site of several thiol-disulfide oxidoreductases. They show low pK_a_ values and a variety of E°′ - however, their E°′ values do not reflect the E°′ of the enzyme they are derived from [37]. Restricting the conformational independence of these small proteins by cyclization results more strained disulfides, thus higher values of E°′; accordingly, these molecules can be more efficient folding catalysts then the linear ones. Upon changing the identity of intervening residues in the CXXC motif, proteins can be obtained with different folding activity corresponding the E°′ and pK_a_ value of the folding enzymes they imitate [38]. Photoactive CXXC peptides also exist. These molecules are cyclized with azobenzene, which changes the conformation and consequently, the reduction potential with light exposition [39].

Besides the identity, the number of the amino acid residues in the C(X)_n_C motif might be also modified. The active site has also been modeled with CXC-containing peptides, which include only one intervening residue between the two Cys. Upon oxidation, the molecule forms a strained 11-membered disulfide-bonded ring and have an E°′ value similar to PDI - however, the rate of pK_a_ of the active site Cys is higher then that of PDI [40].

Other small molecule folding catalysts were designed by using the advantageous properties of PDI without modeling the active site of the enzyme. Although one thiol group is enough for catalysis, the presence of two Cys groups enhances the overall effectiveness of the oxidative folding. The most prevalent dithiol used as oxidative folding catalyst is (±)-*trans-*1,2-bis(mercaptoacetamido)-cyclohexane (BMC), which has an E°’ and first-thiol pK_a_ value close to that of PDI [41]. Indeed, BMC effectively catalyses the formation of the four-disulfide containing native ribonuclease A *in vitro* from its scrambled analogue with random distribution of disulfide bonds. The monothiol analogue of BMC – however in less extent – also shows unscrambling activity. BMC is an effective folding catalyst *in vivo* as well – addition to the growth medium of *S. cerevisiae* increases the yield of the secretion of native, disulfide containing *Schizosaccharomyces pombe* acid phosphatase.

Aromatic thiols are also efficient foldases [42]. They have low thiol pK_a_ values respect to their aliphatic counterparts, which ensures a high reactivity for thiol-disulfide interchange reactions at physiological pH. The thiol pK_a_ can be tuned by changing the substituent on the aromatic ring. A typical representative of the group is the monothiol 4-mercaptobenzeneacetate, whose pK_a_ value (6.6) is similar to that of PDI, is 5–6 times more effective then the commonly used glutathione redox buffer. Among the dithiol components of the group even more active molecules can be found.

The diselenide analogue of oxidized glutathione - GSeSeG – has also been shown to act as an effective oxidant during protein folding [43]. GSeSeG can not only replace GSSG in a standard disulfide containing redox buffer, but works with much higher efficiency compared with its disulfide analogue. Selenols have lower pK_a_ values then thiols, which extends their advantageous kinetic properties to acidic pH. They have enhanced reactivity in both oxidized and reduced forms, although the diselenide bond is more stabile than the disulfide bond. Another organic selenide, selenocystamine is also a commercially available folding catalyst; however exhibits lower activity than GSeSeG, which become even more pronounced under acidic conditions. Nevertheless, besides their advantageous kinetic properties in protein folding, it is notable that selenide derivatives often exhibit high toxicity [44].

The thiol-specific oxidant, dipyridyl disulfide (DPS) was lately shown to affect directly the Ero1-PDI pathway [45]. DPS was able to rescue the *ero1-1* mutant from its temperature sensitivity and the growth defect under anaerobic conditions.

Besides the above enumerated standard redox-active chemicals, non-redox-active small molecules were also shown to assist in disulfide bond formation. This class of compounds - called chemical chaperones - was shown to reverse the intracellular retention of several different misfolded proteins thus were used efficiently in conformational diseases. Among these molecules the most known are glycerol and other polyols, DMSO, trimethylamine-N-oxide and trifluoroethanol. All of them were shown to increase the rate of *in vitro* protein refolding with nonspecific manners, e.g. by stabilizing native-like folding intermediates, by increasing the kinetics of oligomeric assembly, or by inducing the conformational-order in the fully reduced polypeptide [46–47].

## 6. Concluding remarks

Oxidative protein folding is mediated by an electron relay system chiefly composed by luminal proteins of the ER. However, low molecular weight electron acceptors can also contribute to the process. Their participation has been evidenced in bacteria and is suggested also in eukaryotes. Several compounds have been designed, synthesized and used effectively as folding catalysts. Recent observations show that endogenous molecules – principally water- and lipid-soluble antioxidant vitamins – can fulfill the same role. The thiol oxidant effect of dehydroascorbic acid has been demonstrated in *in vitro* systems, and some *in vivo* findings support its involvement in the oxidative folding. The effects of these vitamins on oxidative folding can contribute to their *in vivo* action. Further work is needed for the mapping of the redox network composed by the small electron carrier compounds in the ER lumen.

## Figures and Tables

**Table 1.** The standard redox potentials of small-molecule catalysts of oxidative protein folding.

***Compounds***	***E°' (mV)***
Linear CXXC (active-site sequence of Trx)	−190
Cyclic CXXC (active-site sequence of PDI)	−130
Photoactive CXXC *cis*/*trans* (active-site sequence of Trx-reductase)	−147 (*cis*) / −201(*trans*)
CXC-containing peptides (CGC)	−167
Aromatic thiols: (1.) R = CH_2_COOH; (2.) R = SO_3_H	−170 (1.) / −220 (2.)
GSeH	−407
Selenocystamine	−348
BMC	−240
GSH	−250
ascorbate / dehydroascorbic acid	80
tocopherol / tocopheryl radical	480
vitamin K / vitamin K epoxide	303
dipyridyl-disulfide / dipyridyl-dithiol	147

Catalysts acting by thiol/disulfide exchange are characterized by redox potentials similar to that of PDI. Prooxidant agents (the last four rows) promoting disulfide formation by indirect mechanisms exhibit high standard redox potentials.

## List of abbreviations:

BMC: (±)-*trans-*1,2-bis(mercaptoacetamido)cyclohexane;
DMSO: dimethyl sulfoxide;
DPS: dipyridyl disulfide;
Dsb: disulfide bond formation proteins;
ER: endoplasmic reticulum;
Ero1: endoplasmic reticulum oxidoreductase 1;
ERP72: endoplasmic reticulum protein 72;
FAD: flavin adeninde dinucleotide;
FMN: flavin-mononucleotide;
GLO: gulonolactone oxidase;
GRP78: glucose regulated protein 78;
GRP94: glucose regulated protein;
GSeSeG: diselenide analogue of oxidized glutathione;
GSH: reduced glutathione;
GSSG: oxidized glutathione;
H_2_O_2_: hydrogen peroxide;
PDI: protein disulfide isomerase;
ROS: reactive oxygen species;
UPR: unfolded protein response;
VKORC1: vitamin K1 2,3-epoxide reductase subunit 1.
